# Natural products as metabolic modulators to enhance cancer immunotherapy: reprogramming the tumor microenvironment

**DOI:** 10.3389/fimmu.2025.1740644

**Published:** 2026-01-14

**Authors:** Xiaoyu Fan, Shushu Guo, Wanfang Li, Yingyin Wang, Jie Bao, Jiuming He, Hongtao Jin

**Affiliations:** 1 New Drug Safety Evaluation Center, Institute of Materia Medica, Chinese Academy of Medical Sciences & Peking Union Medical College, Beijing, China; 2 School of Clinical Pharmacy, Shenyang Pharmaceutical University, Shenyang, China; 3 Beijing Union-Genius Pharmaceutical Technology Development Co. Ltd., Beijing, China; 4 State Key Laboratory of Bioactive Substance and Function of Natural Medicines, Institute of Materia Medica, Chinese Academy of Medical Sciences & Peking Union Medical College, Beijing, China; 5 Beijing Key Laboratory of Key Technologies for Preclinical Research and Development of Innovative Drugs in Pharmacokinetics and Pharmacodynamics, Beijing, China

**Keywords:** cancer immunotherapy, immune metabolism regulation, metabolic reprogramming, natural products, tumor microenvironment

## Abstract

Cancer remains a major global health challenge, and although immunotherapy has achieved remarkable breakthroughs, its efficacy is often limited by tumor-induced immunosuppression within the tumor microenvironment (TME). Emerging evidence indicates that metabolic reprogramming plays a pivotal role in shaping the TME and regulating antitumor immune responses. Targeting tumor and immune cell metabolism has therefore become a promising strategy to enhance the effectiveness of cancer immunotherapy. This review first summarizes the metabolic reprogramming that occurs within the TME, including alterations in glucose, lipid, and amino acid metabolism in tumor cells, as well as the metabolic adaptation of immune cells. We then highlight recent advances in natural products that modulate key metabolic pathways and their potential to reshape the immunosuppressive TME. Special emphasis is placed on natural compounds that not only inhibit tumor cell metabolism but also restore the metabolic fitness of immune cells, thereby improving antitumor immunity. In addition, advances in delivery strategies, including nanocarrier-based and stimuli-responsive systems, are reviewed for their roles in improving the bioavailability, stability, and tumor targeting of natural metabolism-regulating agents. Finally, we discuss the current status and challenges of translating natural metabolism-regulating agents into clinical applications, including issues of dose optimization, safety evaluation, and patient selection. Despite these hurdles, precision targeting of metabolic pathways, interdisciplinary collaboration, and the discovery of novel compounds—particularly immune-sensitizing agents derived from traditional medicine—are expected to accelerate progress. Collectively, natural products represent promising adjuvant strategies for cancer immunotherapy, with great potential to overcome current therapeutic limitations and improve clinical outcomes.

## 1 Introduction

For decades, cancer has remained one of the most significant threats to global public health, with incidence and mortality rates continuing to rise worldwide. Despite advancements in early detection and treatment modalities, cancer imposes a substantial burden on healthcare systems and severely impacts patient quality of life (1). Immunotherapy has emerged as a transformative approach in cancer treatment, offering unprecedented clinical benefits in various malignancies. Strategies such as immune checkpoint blockade, adoptive cell transfer, and cancer vaccines have demonstrated durable responses and long-term survival in subsets of patients (2, 3). However, the efficacy of immunotherapy remains limited by several challenges, including tumor immune evasion (4), an immunosuppressive tumor microenvironment (5), and the low immunogenicity of certain cancer types (6). These limitations underscore the urgent need for novel strategies to enhance the effectiveness of immunotherapy and broaden its clinical applicability.

Targeting cancer metabolic abnormalities has been recognized as a promising strategy to remodel the tumor microenvironment and enhance antitumor immune response (7). Tumor cells undergo profound metabolic reprogramming to support their rapid proliferation, survival, and immune evasion. These metabolic alterations, including increased glucose uptake coupled with abundant lactate secretion, as well as alterations in the tricarboxylic acid cycle (TCA cycle) - has a major impact on the immunological tumor microenvironment (TME) (8). Modulating these aberrant metabolic pathways can disrupt the tumor’s protective microenvironment and restore antitumor immunity (8). Current studies have demonstrated that metabolic interventions can increase immune cell infiltration, enhancing antitumor T cells, suppressive myeloid cells and synergize with immune checkpoint blockade therapies (9, 10). Therefore, targeting tumor metabolism may offer the potential to overcome the current limitations of immunotherapy and expand its clinical efficacy in a wider range of malignancies.

Understanding the metabolic alterations in tumor cells has led to the exploration of targeting these pathways as a therapeutic strategy. However, translating these insights into effective clinical therapies has proven challenging. Although Otto Warburg’s theory of aerobic glycolysis laid the foundation for tumor metabolism research, this metabolic pathway has not been successfully exploited in the clinic, largely due to the adverse side effects and limited efficacy of 2-deoxyglucose, which inhibits glycolysis. Farber’s pioneering use of antifolate drugs established cancer chemotherapy, and another folate antagonist, methotrexate, remains a key component of childhood acute lymphoblastic leukemia (ALL) treatment, with a cure rate of up to 90% when combined with l-asparaginase. Additionally, several antimetabolites, particularly those targeting nucleotide metabolism, have been approved for clinical use. Moreover, drugs targeting mutant isocitrate dehydrogenase in acute myeloid leukemia (AML) have marked a milestone in precision cancer metabolism therapy, demonstrating the potential effectiveness of metabolic treatment (10–12). Despite successes in tumor metabolism-targeted therapies, challenges such as significant toxicity and limited therapeutic windows remain, highlighting the need for more selective and safer agents. As research into the tumor immune microenvironment progresses, the interplay between metabolism and immune cells has garnered renewed attention (8). Future research may focus more on improving the tumor immune microenvironment by regulating metabolism, thereby enhancing the efficacy of tumor immunotherapy.

Due to its extensive clinical application experience and wide accessibility, traditional Chinese Medicine (TCM) plays a crucial role in cancer treatment in China (13). It has been reported that TCM can alleviate the side effects associated with conventional therapies and improve the prognosis of patients with various types of cancer (14, 15). Besides, many natural compounds have multi-target activities, which make them have the potential to regulate multiple metabolic pathways simultaneously or regulate both immune and metabolic related pathways, thereby synergistically enhancing their therapeutic effects. For instance, curcumin has demonstrated dual functions in cancer therapy by regulating both tumor metabolism and immune responses. It inhibits tumor cell proliferation through targeting key enzymes involved in the ROS metabolic pathway, while simultaneously exerting immunomodulatory effects by modulating immune cell activities and cytokine expression, including the suppression of TNF-α production and inhibition of dendritic cell maturation (16–18). Given these promising effects, these natural agents, with their lower toxicity profiles and broad spectrum of action, may provide a valuable strategy for overcoming the limitations of current tumor metabolism-targeted therapies.

**Figure 1:**
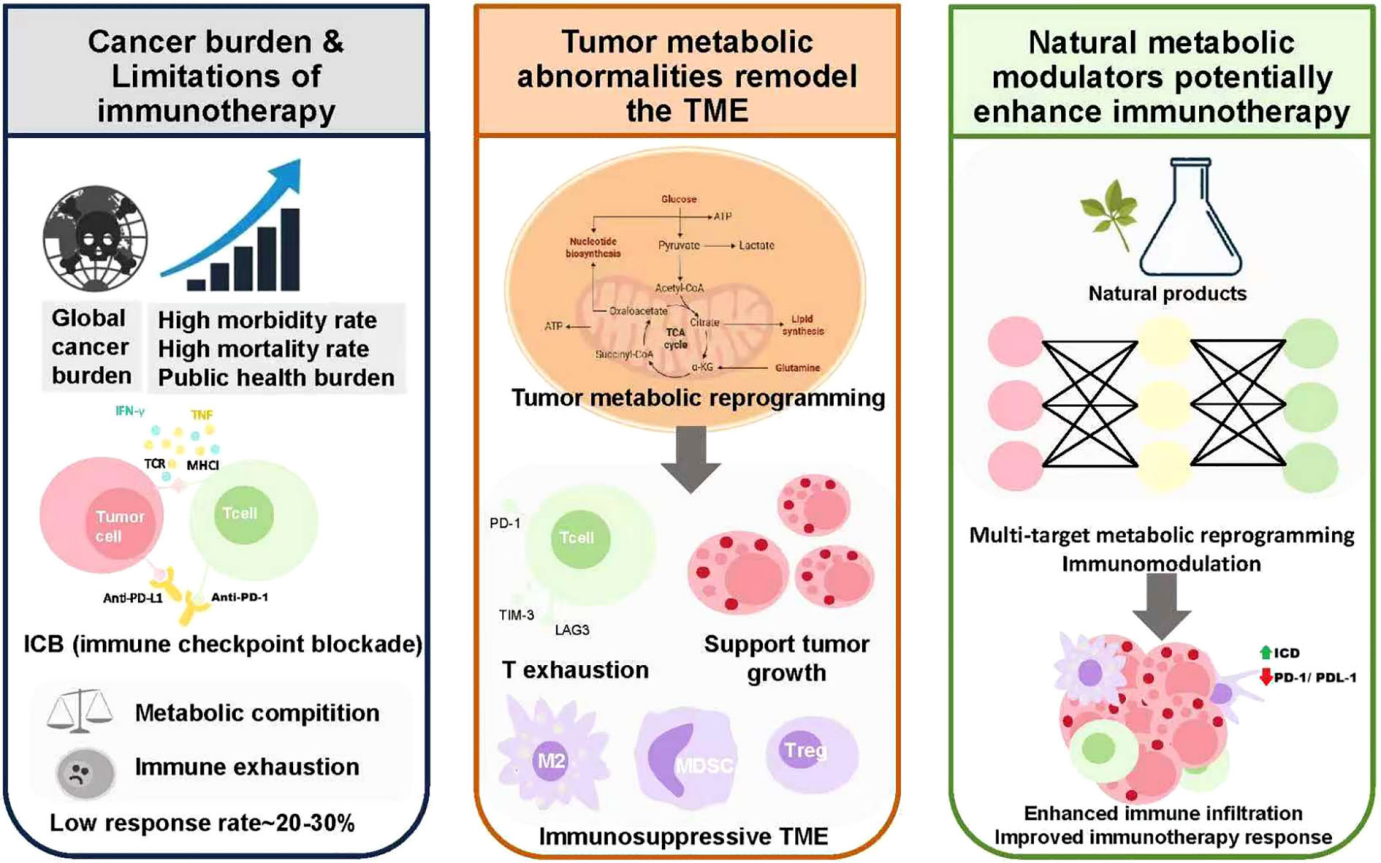
Targeting tumor metabolism with natural products to enhance immunotherapy.

Above all, targeting cancer metabolic abnormalities has been recognized as a promising strategy to remodel the tumor microenvironment and enhance antitumor immune responses. Meanwhile, traditional Chinese medicine, with its multi-target regulatory properties and immunomodulatory potential, has emerged as an important resource for the development of cancer immuno-sensitizers. Based on these insights, this study systematically reviews the latest advances in tumor metabolic reprogramming, the regulatory role of natural products in tumor metabolism, and emerging delivery and analysis technologies. It further explores the potential of natural metabolic modulators in improving the tumor immune microenvironment and enhancing immunotherapy sensitivity. These perspectives aim to provide new strategies and conceptual support for optimizing cancer immunotherapy by coordinating metabolic and immune regulation (**Figure 1**).

## 2 Metabolic reprogramming of the tumor immune microenvironment

**Figure 2:**
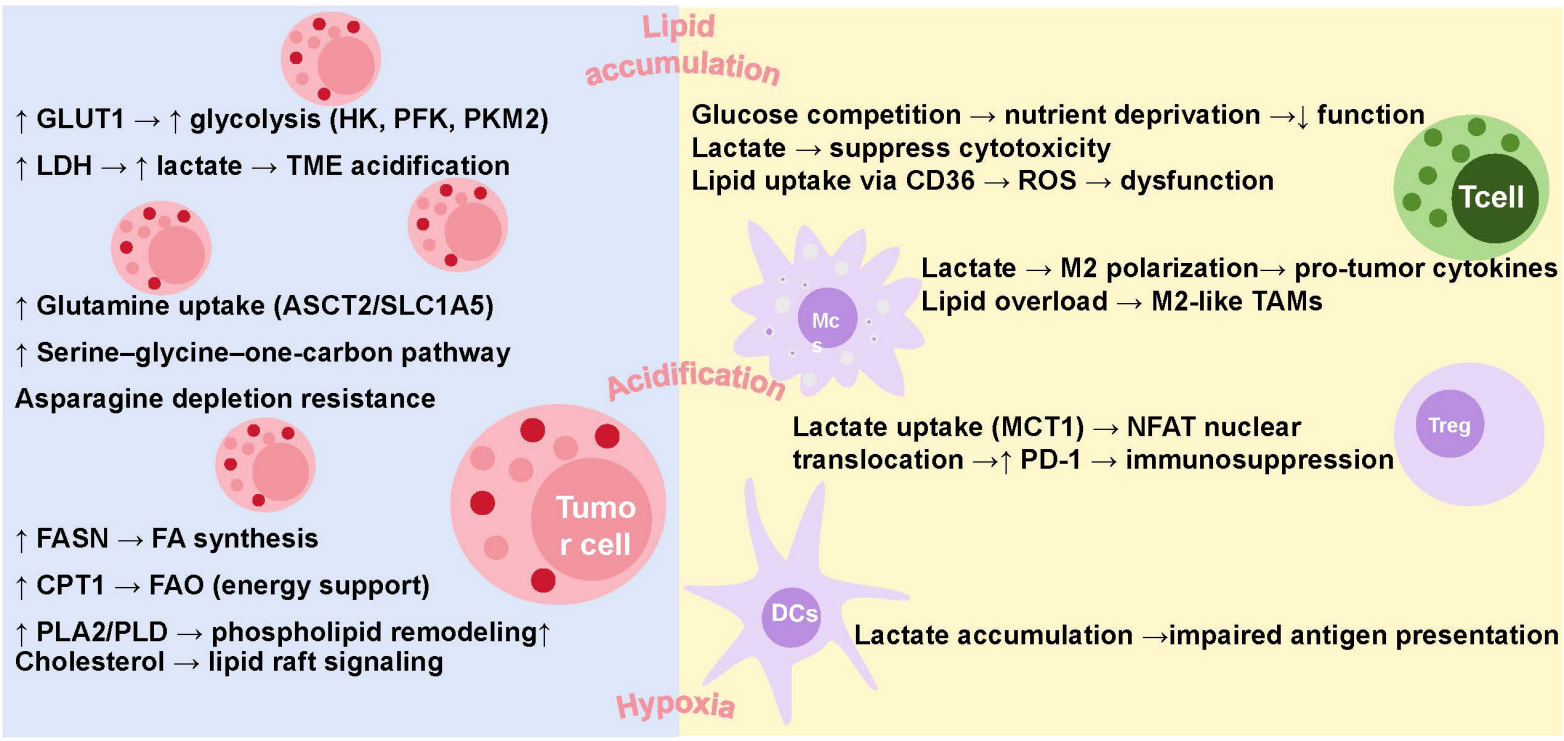
Metabolic reprogramming of glucose, amino acids, and lipids in the tumor immune microenvironment.

Glucose, lipid, and amino acid metabolism constitute the core energy and biosynthetic pathways essential for cellular function. Although often described independently, these metabolic processes are highly interconnected, with dynamic cross-regulation ensuring metabolic flexibility under stress. Within the tumor microenvironment, this cross-talk is further complicated by the coexistence of tumor and immune cells, both of which undergo metabolic reprogramming. Tumor and immune cells engage in metabolic competition and mutual modulation, shaping a metabolic landscape that profoundly influences tumor progression and immune responses. The following sections will provide an overview of the effects of the three major energy metabolic pathways on immune cells and tumor cells (**Figure 2**).

### 2.1 Warburg effect and glycolysis in cancer

Glucose metabolism is a central pathway for cellular energy production and biosynthesis. Through glycolysis, cells rapidly convert glucose into pyruvate, generating ATP and metabolic intermediates (19). Under aerobic conditions, pyruvate typically enters the mitochondria for oxidative phosphorylation (OXPHOS). However, cancer cells exhibit enhanced glycolysis even in the presence of oxygen, a phenomenon known as the Warburg effect (20). This metabolic reprogramming promotes tumor growth, enhances survival under hypoxic conditions, and contributes to resistance against various therapies (21, 22). The reprogramming of the glycolytic pathway involves not only changes in metabolic flux but also the regulation of the expression and activity of key enzymes. Among these, hexokinase (HK), phosphofructokinase (PFK), and pyruvate kinase M2 (PKM2) function as rate-limiting enzymes that directly determine the speed and direction of glycolysis. Regulation of these enzymes has been shown to play a critical role in the initiation and progression of various cancers, as well as in angiogenesis, immune evasion, and drug resistance (23, 24).

HK catalyzes the first irreversible step of glycolysis by phosphorylating glucose to glucose-6-phosphate. Studies have shown that HK2 is upregulated in various tumors, promoting cancer cell proliferation, invasion, and metastasis (25, 26). HK2 expression is elevated in both primary and metastatic lung cancers, and its upregulation can be suppressed by KRas deletion or overexpression of p53 and KEAP1 (27). Loss of HK2 inhibits migration and invasion of non-small cell lung cancer (NSCLC) cells, enhances cisplatin sensitivity, and reduces lung metastasis (28). In pancreatic ductal adenocarcinoma (PDAC), reduced HK2 expression inhibits VEGF-A signaling, thereby suppressing tumor growth and metastasis in mice (29). Glucose analogs such as 2-deoxy-D-glucose (2-DG) and 3-bromopyruvate (3-BrPA) can inhibit HK2, block glycolysis, and enhance the anticancer effects of other drugs, however, their toxicity limits clinical application (30, 31).

Phosphofructokinase-1 (PFK-1), another key rate-limiting enzyme of glycolysis, exists in three isoforms including PFKM, PFKL, and PFKP. Based on current researched, high PFKP expression is negatively correlated with overall survival in various cancers, including cervical, liver, and lung cancers (32). Tat activating regulatory DNA-binding protein (TARDBP) suppresses PFKP expression through miR-520, thereby inhibiting glycolysis and tumor growth in hepatocellular carcinoma (HCC) (33). Moreover, PFKP expression is upregulated in hepatocellular carcinoma, contributing to HCC proliferation and stemness maintenance (34). In brain tumors, EGFR mediates phosphorylation of PFKP at Y64, activating the PI3K signaling pathway and promoting proliferation (35).

The final step of glycolysis is catalyzed by pyruvate kinase (PK), which converts phosphoenolpyruvate (PEP) to pyruvate and generates ATP. Mammalian PK has four isoforms with distinct tissue distributions, PKM1 is predominantly expressed in normal adult cells favoring oxidative phosphorylation, while PKM2 is highly expressed in rapidly proliferating tumor and non-malignant cells, favoring glycolysis (36). PKM2 is significantly upregulated in colorectal cancer (CRC), inflammatory bowel disease, and intrahepatic cholangiocarcinoma (ICC), and is associated with higher tumor necrosis, angiogenesis, and advanced stages (37). High PKM2 expression is also linked to poor prognosis in multiple myeloma patients (38). Moreover, inhibiting PKM2 can enhance drug sensitivity; for instance, its interaction with CD44 promotes Thr105 phosphorylation, reducing PKM2 activity, elevating ROS, and sensitizing CRC cells to cisplatin. CD44 deficiency shifts glycolysis toward the TCA cycle, further increasing ROS and drug efficacy (39). Under hypoxia, IGF-1/IGF-IR promotes HIF-1α binding to NF-κB p65/RelA and the PKM2 promoter, while miR-148a/152 suppression upregulates PKM2. This induces PKM2 nuclear translocation, where it acts as a kinase to regulate VEGF expression and drive tumor angiogenesis (40).

Lactate dehydrogenase (LDH) has gained increasing attention for its significance in cancer. Elevated serum LDH levels, particularly in melanoma patients, are often associated with high tumor burden, poor prognosis, and unfavorable therapeutic outcomes. Although LDH is primarily used clinically as a prognostic biomarker reflecting tumor burden, growing evidence suggests that it also plays functional roles in tumorigenesis and progression (41). Pyruvate, the end product of glycolysis, is reduced to LDH to regenerate NAD^+^, thereby maintaining a high-flux glycolysis metabolic process. This metabolic reprogramming leads to the continuous production and accumulation of large amounts of lactate, which in turn causes acidification of the TME, promoting cancer cell growth, migration, and angiogenesis (42, 43). Moreover, Monocarboxylate transporters (MCT1 and MCT4), which regulate lactate transport across the cell membrane, are overexpressed in multiple cancers and help maintain the tumor microenvironment (44). Studies have shown that MCT4 knockdown or MCT inhibition reduces lactate uptake and migration in PDAC cells (45). Early studies also indicate that targeting MCT1 can suppress cancer cell growth and proliferation, and several small-molecule MCT1 inhibitors have been reported (46).

In addition, glucose transport plays a crucial role in regulating tumor glycolysis. Glucose transporters (GLUTs) are commonly expressed on cell membranes, with GLUT1 being the most widely distributed isoform. Meta-analyses have shown that GLUT1 is frequently overexpressed in various solid tumors and is associated with poor prognosis (47). Moreover, the GLUT1 inhibitor BAY-876 has been reported to significantly reduce glucose uptake, cell viability, and metabolic activity in head and neck squamous cell carcinoma (HNSCC), while also inducing apoptosis (48).

In summary, the upregulation of key glycolytic enzymes such as HK, PFK, and PKM2, as well as LDH and lactate transporters MCT1 and MCT4, along with increased expression of the glucose transporter GLUT1, enhances glycolytic activity in tumors, contributes to an acidic TME, and promotes cancer cell invasion and migration.

### 2.2 Amino acid metabolism reprogramming in cancer

To support rapid proliferation under nutrient-deprived microenvironments, tumor cells undergo extensive metabolic reprogramming, often adopting the “use of opportunistic modes of nutrient acquisition” strategy (49). In addition to altered glucose metabolism, dysregulated amino acid metabolism has emerged as a key hallmark of cancer. Amino acids serve not only as building blocks for protein synthesis but also as vital sources of carbon and nitrogen, contributing to energy production, redox balance, and the regulation of multiple signaling pathways—including mTORC1, MYC, and KRAS—that drive tumor progression. Furthermore, certain amino acid-derived metabolites, such as α-ketoglutarate (α-KG), acetyl-CoA, NAD^+^, and S-adenosylmethionine (SAM), modulate gene expression through epigenetic mechanisms (50). Collectively, these processes form a complex metabolic network that provides a rationale for targeting amino acid metabolism in cancer therapy. Compared with normal cells, tumor cells exhibit enhanced dependence on amino acids, with heterogeneous requirements across different cancer types (51). The heterogeneous dependence of tumor cells on specific amino acids makes amino acid metabolism a key target for anti-cancer therapy. Restricting essential amino acids may starve cancer cells, while supplementing certain amino acids in specific contexts may enhance therapeutic efficacy, highlighting the complexity and precision required in metabolic interventions.

Among amino acid-targeted therapies, asparagine (Asn) remains the most well-established and clinically effective target for amino acid depletion therapy. In pediatric ALL, bacterially derived asparaginase (ASNase) markedly improved cure rates (52). Glutamine is the most abundant amino acid in plasma and the most rapidly consumed in tumors (53). Tumor cells show “glutamine addiction”, and its deficiency often arrests cancer cells in the S phase (54). ASCT2 (SLC1A5) is the major glutamine transporter in tumors, and studies have found that its high expression is associated with poor prognosis in a variety of tumors (55). SLC1A5 is a key target in glutamine metabolism, but current inhibitors show limited specificity. While V-9302 and new monoclonal antibodies demonstrate promise in preclinical models, especially in colorectal cancer, effective and selective inhibitors for broader clinical use remain scarce (56). Serine is a central metabolite involved in the synthesis of proteins, amino acids, and nucleotides. Cancer cells fulfill their serine demand either through exogenous uptake or via *de novo* synthesis, thereby fueling the serine–glycine–one-carbon (SGOC) metabolism pathway. This pathway is critical for tumorigenesis and holds substantial clinical significance (57). Preclinical studies have shown that dietary restriction of serine and glycine significantly inhibits tumor proliferation in mouse models of intestinal cancer and lymphoma (58). Moreover, serine deprivation can induce ROS production, activate the AMPK pathway, and remodel the cytoskeleton to enhance cancer cell motility. Inhibition of serine/glycine uptake or knockdown of SHMT1 suppresses the migration of lung adenocarcinoma cells (59, 60). Moreover, other amino acids, such as arginine, methionine, and others, have also been widely implicated in tumor metabolic reprogramming (50, 61). Notably, amino acid supplementation may exhibit anti-tumor effects in specific contexts. Ishak Gabra et al. reported that glutamine supplementation inhibited melanoma growth by modulating epigenetic reprogramming (62). In line with this, histidine supplementation increased the sensitivity of leukemia xenograft models to methotrexate (63).

Dysregulated amino acid metabolism is crucial for tumor growth, offering both metabolic support and regulatory control. The varying amino acid dependencies across cancers highlight the potential of targeting these pathways. Strategies involving amino acid depletion or supplementation have shown context-dependent anti-tumor effects, underscoring the need for precise metabolic interventions in cancer therapy.

### 2.3 Lipid metabolic reprogramming in cancer

Lipids represent a structurally diverse group of biomolecules, classically categorized into fatty acids (FAs), glycerol-based lipids (including neutral triacylglycerols and phosphoglycerides), and non-glycerol-based lipids (such as sterols and sphingolipids). Except for severing as an energy source via fatty acid oxidation (FAO), lipids are essential for maintaining cellular membrane integrity and mediating a wide array of signal transduction processes (64). In the tumor microenvironment, lipid metabolism undergoes profound reprogramming to support the demands of uncontrolled proliferation and survival (65). This metabolic adaptation ensures a continuous supply of lipid precursors for membrane biosynthesis, facilitates ATP production through enhanced fatty acid oxidation, and generates lipid-derived signaling molecules that function as second messengers in multiple oncogenic pathways. Moreover, dysregulated lipid metabolism influences key tumor-associated biological processes, including endoplasmic reticulum (ER) stress responses and ferroptosis (66, 67). As such, inhibition of lipid biosynthetic pathways has been recognized as a potential therapeutic strategy for impairing tumor growth and viability.

Fatty acid metabolism, including *de novo* synthesis and FAO plays a crucial role in supporting tumor growth, progression, and therapy resistance. Fatty acid synthase (FASN), a key enzyme in *de novo* lipogenesis, catalyzes the synthesis of long-chain fatty acids from acetyl-CoA and malonyl-CoA precursors. Upregulation of FASN has been widely reported across multiple malignancies, including colorectal, prostate, ovarian, gastrointestinal, lung cancer, et al., (68). Notably, hypoxic TMEs induce the overexpression of hypoxia-inducible factor 1-alpha (HIF-1α), which in turn promotes the transcription of lipid-associated genes, such as lipid transporters, FASN, and sterol regulatory element-binding protein 1 (SREBP1). This coordinated upregulation facilitates enhanced lipid uptake, transport, and synthesis, ultimately contributing to the proliferation and migration of cancer cells, as observed in HCT-116 colorectal cancer cells (69). In addition to *de novo* lipogenesis, FAO is often upregulated in tumors to meet the high energy demands of rapidly proliferating cells. FAO is regulated by a series of fatty acid oxidases and is closely linked to cancer cell invasiveness (70). Carnitine palmitoyltransferase 1 (CPT1), the rate-limiting enzyme in FAO, facilitates the mitochondrial transport of long-chain fatty acids for β-oxidation. Inhibition of CPT1-mediated FAO has been shown to sensitize tumor cells to chemotherapy- or immunotherapy-induced cell death, highlighting its potential as a therapeutic target (71). Moreover, CD36, a membrane-associated lipid transporter, promotes cancer cell migration by enhancing fatty acid uptake and activating FAO (72).

Phospholipids are important components of cell membranes and play an important role in maintaining cell membrane structure and normal function as well as regulating signal transduction and cell cycle. Alterations in phosphoglyceride metabolism in cancer can be attributed to dysregulation of rate-limiting enzymes involved in phosphoglyceride metabolism, which are critical for tumor growth. Phospholipase A_2_ (PLA_2_) is a phospholipid metabolizing enzyme that releases free fatty acids, mostly arachidonic acid, and lysophospholipids, which contribute to the development of the TME, promoting immune evasion, angiogenesis, tumor growth, and invasiveness (73). Studies have reported that in stage II colorectal cancer, patients with PLA2-negative tumors have significantly longer disease-free survival, suggesting that PLA2 may be a prognostic predictor for colorectal cancer (74). Moreover, In colorectal cancer, inhibition of PLD activity suppresses PA production and inactivates mTOR signaling, thereby interfering with cancer cell proliferation (64). In addition, phospholipid metabolites are also essential for the biological functions of cells. Arachidonic acid is an important phospholipid metabolite that is associated with carcinogenesis. PIK3CA mutant tumor cells transmit oncogenic signals and result in malignant transformation of intestinal epithelial cells (IECs) via paracrine exosomal arachidonic acid (AA)-induced H3K4 trimethylation (75). Other studies have found that arachidonic acid metabolism inhibitors synergize with immune checkpoint inhibitors to inhibit the growth of ARID1A-deficient colorectal cancer by enhancing CD8+ T cell activity and inhibiting vasculogenic mimicry (76).

Besides, cholesterol also plays an important role in cancer development. Both clinical and experimental studies have found that hypercholesterolemia and a high-fat, high-cholesterol diet can affect cancer development. External cholesterol can directly activate the oncogenic Hedgehog pathway, and internal cholesterol can induce mTORC1 signaling (76). Cholesterol is not only a precursor of hormones such as vitamin D, progesterone, and estrogen, but also participates in the formation of lipid rafts, which are important platforms for the regulation of multiple oncogenic signaling pathways (77).

### 2.4 Effects of metabolism on immune cells in the TME

Tumor-infiltrating immune cells have dual characteristics, some of which have the function of inhibiting tumor proliferation and metastasis, such as CD8+ cytotoxic T cells, natural killer (NK) cells, M1 macrophages, dendritic cells (DCs), etc., while others may be beneficial to tumor development, including Treg cells, M2 macrophages (78). The TME, characterized by nutrient deficiency, lactate accumulation, lipid deposition, and hypoxia, significantly impairs immune function and weakens anti-tumor responses (79). In addition, studies have found that certain metabolites in the TME can suppress immune activity. In response to these challenges, immune cells also adjust their metabolic processes to resist tumor progression (80).

The proliferation of tumor cells and immune cells depends on glycolysis, and tumor cells compete for glucose, which makes immune cells lack nutrients and unable to perform their functions (81). Furthermore, the expression of the key glycolytic enzyme PFKFB3 is upregulated and mediates increased PD-L1 expression by activating the nuclear factor κB signaling pathway, thereby inhibiting the anti-tumor activity of cytotoxic CD8+ T lymphocytes (82). In addition, metabolic byproducts produced during glycolysis, such as lactate, significantly inhibit anti-tumor immune responses. Lactic acid interferes with the differentiation of monocytes and DCs, thereby hindering antigen presentation and immune priming (83). In regulatory T cells, lactate is taken up by MCT1 and activates the nuclear translocation of NFAT (nuclear factor of activated T cells), thereby upregulating the expression of programmed cell death protein 1 (PD-1) and enhancing its immunosuppressive activity (84). Lactic acid promotes the polarization of tumor-associated macrophages (TAMs) into a pro-tumor M2-like phenotype, and this lactate-induced M2 polarization facilitates pituitary adenoma invasion by enhancing CCL17 secretion (85). Moreover, lactic acid can promote immune evasion by suppressing the function of NK cells through inhibition of nuclear factor of activated T cells (NFAT) in NK cells (85).

When glucose availability is limited within the TME, tumor-infiltrating immune cells may undergo metabolic reprogramming to sustain their functional capacity, notably by increasing the uptake and oxidation of fatty acids (FA). Currently, the impact of lipid metabolism on immune cell function has been extensively discussed. For instance, lipid accumulation in the TME drives CD8^+^ T cell dysfunction via the CD36 receptor. CD36 mediates the uptake of oxidized lipids, leading to lipid peroxidation and p38 kinase activation, which impair T cell function (86). Moreover, intracellular lipid accumulation or supplementation with exogenous fatty acids can trigger the generation of reactive oxygen species (ROS) and the secretion of immunosuppressive cytokines, thereby facilitating the polarization of macrophages toward a pro-tumorigenic M2 phenotype, which in turn enhances tumor cell invasiveness and metastatic potential (87). In addition, studies have reported that abnormal lipid accumulation in the TME leads to decreased antigen presentation ability of DCs and anti-tumor ability of T cells (88).

In the TME, tumor cells have significantly higher metabolic activity than normal cells and competitively inhibit the uptake of amino acids by immune cells, thereby inhibiting antitumor immune activity (50).

For example, increased tumor uptake of glutamine similarly limits its availability to T cells, impairing the proliferation and activation of especially effector T cells. During glutamine deprivation, Teff cells show decreased c-MYC protein expression, growth restriction, and impaired immune function (89). In addition, glutamine deprivation promotes the differentiation of Treg cells through AMPK-mTORC1 signaling pathway, thereby reducing the immune function of Teff cells (90). Moreover, Tryptophan is an essential amino acid that is only obtained through diet and is rapidly depleted in the TME due to increased consumption and catabolism by tumor cells. Tumor cells metabolize tryptophan to kynurenine, which is a ligand for the aryl hydrocarbon receptor (AHR). AHR activation in CD4^+^ T cells promote their differentiation into immunosuppressive regulatory T cells (Tregs), while kynurenine also induces PD-1 expression on CD8^+^ T cells, inhibiting their cytotoxic function (91). Based on current studies, arginine deprivation further compromises T cell function by reducing mTORC1 activity, leading to an increased proportion of memory-like T cells while diminishing effector functions and immune surveillance (92). Additionally, M2-like TAMs upregulate arginase 1 (Arg1), enhancing arginine metabolism and further depleting extracellular arginine, thereby exacerbating immunosuppression (93).

In summary, the harsh metabolic landscape of the TME, characterized by nutrient competition, accumulation of immunosuppressive metabolites, and dysregulated lipid and amino acid metabolism, severely impairs the function and fate of infiltrating immune cells, ultimately promoting tumor immune evasion and progression. Understanding these metabolic interactions offers important therapeutic opportunities to restore antitumor immunity and improve cancer treatment outcomes.

### 2.5 Integrated immunometabolic targets and technological advances in the tumor microenvironment

Metabolic reprogramming drives the formation of an immunosuppressive TME through multiple mechanisms, thereby promoting cancer progression and diminishing the efficacy of anticancer immunotherapies. Within the TME, nutrient scarcity—particularly of glucose and amino acids—combined with the accumulation of immunosuppressive metabolites such as lactate, adenosine, and kynurenine creates a metabolic niche that impairs the function of antitumor immune cells. However, traditional metabolic profiling approaches—such as tissue homogenization coupled with gas chromatography- mass spectrometry (GC-MS) or liquid chromatography-mass spectrometry (LC-MS)—despite their success in identifying tumor-specific metabolic signatures, discovering prognostic biomarkers, and revealing adaptive metabolic responses under therapeutic stress limited (94). These methods disrupt cellular spatial architecture, overlook cell–cell interactions, and perturb the native metabolic state, thus failing to capture the full spatiotemporal heterogeneity of metabolism within the tumor-immune ecosystem.

Recent advances in single-cell sequencing and spatial metabolomics have provided transformative solutions to long-standing challenges in understanding tumor heterogeneity and therapeutic resistance. The advent of single-cell technologies now enables the interrogation of metabolic states at the resolution of individual immune cells within clinical tumor samples, revealing pronounced context-dependent metabolic heterogeneity across immune cell subset (95, 96). In parallel, matrix-assisted laser desorption/ionization mass spectrometry imaging (MALDI–MSI) allows label-free, near–single-cell-resolution mapping of thousands of metabolites directly in intact tissue sections while preserving spatial architecture (97, 98). Application of MALDI–MSI across diverse malignancies—including colorectal, lung, breast, liver, and brain tumors—has uncovered spatially organized metabolic gradients associated with hypoxia, immune infiltration, stromal composition, and therapeutic response, thereby providing mechanistic insights into regional immune suppression and functional divergence of immune cells within the TME (98, 99).

Moreover, the integration of single-cell transcriptomics with spatial metabolomics, together with complementary genomic, proteomic, and epigenomic data, enables a systematic, multi-dimensional reconstruction of tumor–immune interactions. Such integrative approaches reveal how dynamic metabolic remodeling within the TME—including hypoxia, nutrient deprivation, and immunosuppressive metabolite accumulation—reshapes immune cell states and fosters therapeutic resistance. Beyond tumor cell–intrinsic mechanisms such as genomic instability and aberrant signaling, this spatially resolved, single-cell multi-omics framework highlights the critical role of TME-driven immune metabolic reprogramming in mediating resistance to chemotherapy, targeted therapy, and immunotherapy.

## 3 Natural products targeting tumor metabolism

Although a variety of chemotherapeutic and molecularly targeted agents have been developed to inhibit key metabolic enzymes, their clinical application is often limited by modest efficacy, drug resistance, and severe adverse effects. In contrast, natural products have attracted increasing attention as promising candidates owing to their multitarget regulatory properties, relatively low toxicity, and ability to modulate the tumor microenvironment. These compounds exert comprehensive regulatory effects on tumor metabolism, including the modulation of glycolysis, lipid metabolism, and oxidative phosphorylation. **Table 1** provides a summary of representative natural products and their corresponding metabolic target.

**Table 1:** Natural products modulating metabolic enzymes and pathways in cancer.

Target	Natural product	Mechanism of action	Outcome	Reference
HK2	Curcumin	Downregulates HK2 → mitochondrial dysfunction	Apoptosis	(100)
	Epigallocatechin gallate (EGCG)	Disrupts HK2–mitochondria binding → impaired energy metabolism	Apoptosis	(101)
	Arsenic trioxide (As_2_O_3_)	Inhibits HK2 expression and glycolysis	Apoptosis	(102)
GLUTs	Phloretin	Suppresses GLUT2 via HNF6 downregulation + activates p53	Cell cycle arrest & apoptosis	(103)
	Apigenin	Downregulates GLUT1 (via HIF-1α) + reduces VEGF secretion	Anti-proliferative & anti-metastatic	(104)
	Trehalose	Inhibits glucose transport → ATP depletion → AMPK activation	Autophagy	(105)
PKM2	Shikonin	Selectively inhibits PKM2	Decreases glucose uptake & increase lactate production	(109)
	Lapachol	Inhibits PKM2 → reduces ATP; sensitizes cells to mitochondrial proton transporters	Apoptosis and antiproliferative effects	(110)
LDHA	Gossypol	Inhibits LDHA → reduced lactate generation	Apoptosis	(111)
	Oxamate	LDHA inhibitor → reduced lactate synthesis	Apoptosis	(112)
GLUT1, HK2	Genistein	suppresses GLUT1 and HK2 expression by downregulating HIF-1α	Cell cycle arrest, reduces the tumor cell invasive capacity	(107)
HK2, PFK1	Oleanolic acid (OA)	Inhibits HIF-1α, HK2, and PFK1 → reduced glucose uptake and lactate	Suppresses tumor cell growth	(114)
FASN	Luteolin	Downregulates anti-apoptotic proteins	Cell cycle arrest	(115)
ACLY	Curcumin	Inhibits ACLY → reduced acetyl-CoA → impaired lipid synthesis	Suppresses tumor growth	(117)
	Berberine	Inhibits ACLY activity	Inhibits tumor progression & metastasis	(118)
cholesterol	Naringenin	Reduces foam cell formation, intracellular cholesterol, and esterification → enhances cholesterol efflux	Regulates cholesterol metabolism, suppresses tumor progression	(119)
amino acid homeostasis	Huangqin Tang (HQT)	Modulating the PI3K/AKT/mTOR pathway	Balancing proliferation and apoptosis	(120)
	Wu Mei Wan (WMW)	Modulating the PI3K/AKT/mTOR pathway	Suppressing myeloid-derived suppressor cells	(121)
OXPHOS/Mitochondria	Rhein–DCA	Mitochondrial accumulation → inhibits PDK–PDH axis	Disrupts respiratory chain, induces cell death	(126)
	Frondoside A	Downregulates Bcl-2 and survivin → ↑ROS, Cyt c release	Apoptosis	(127)
	Ginsenoside compound K	Activates ROS-mediated mitochondrial apoptosis → Cyt c release + caspase-9/-3 activation	Apoptotic	(128)
PKM2, FASN, OXPHOS, Mitochondria	Resveratrol	inhibits lipid synthesis via suppression of SREBPs+activates sirtuins+ AMPK activation+downregulates PI3K/AKT/mTOR pathway; Down regulating PKM2 via inhibition of mammalian target of rapamycin	cell apoptosis.	(129)

### 3.1 Regulation of glycolysis

As mentioned above, key glycolytic enzymes such as HK, PFK, and PKM2, along with LDH, lactate transporters MCT1 and MCT4, and the glucose transporter GLUT1, are critical for tumor proliferation and metastasis. Current studies have revealed that many natural products exert significant regulatory effects on these proteins and enzymes, thereby contributing to their antitumor activities. Studies have demonstrated that curcumin downregulates HK2, leading to mitochondrial dysfunction and apoptosis in CRC cells (100). EGCG (epigallocatechin gallate, a green tea polyphenol) disrupts the binding of HK2 to mitochondria, thereby impairing energy metabolism and inducing apoptosis (101). Arsenic trioxide (As_2_O_3_) inhibits HK2 expression and glycolysis in cancer cells, triggering apoptosis (102). Besides, phloretin suppresses GLUT2 expression by blocking the transcription factor HNF6 and additionally activates the p53 pathway, promoting cell cycle arrest and apoptosis (103). Apigenin downregulates GLUT1 via inhibition of HIF-1α, leading to reduced VEGF secretion and exhibiting both antiproliferative and antimetastatic effects (104). Trehalose suppresses glucose transport by inhibiting glucose transporter activity, inducing a starvation-like state characterized by ATP depletion. This metabolic stress activates autophagy through AMPK-dependent signaling, thereby exerting anticancer effects (105). Genistein suppresses GLUT1 and HK2 expression by downregulating HIF-1α (106), induces cell cycle arrest, and reduces the invasive capacity of CRC cells (107). Resveratrol inhibits cancer cell metabolism by down regulating pyruvate kinase M2 via inhibition of mammalian target of rapamycin (108). Shikonin selectively inhibits PKM2, decreasing glucose consumption and promotes the release of lactate in MCF-7 and A549 tumor model (109). Similarly, Lapachol is a potent PKM2 inhibitor that inhibits glycolysis and reduces ATP production in melanoma cells. Lapachol also effectively sensitizes cells to mitochondrial proton transporters, promoting apoptosis and antiproliferative effects (110). Gossypol inhibits LDHA, lowering lactate production and glycolytic flux while inducing apoptosis (111). Oxamate, another LDHA inhibitor, induces mitochondrial apoptosis in cancer cells by suppressing lactate production. A study showed that Oxamate exhibits synergistic antitumor effects in preclinical models when combined with metformin or mTOR inhibitors (112). Moreover, in TCM, oleanolic acid (OA), a triterpenoid compound abundant in Oleaceae plants, has emerged as a potential therapeutic candidate for gastric cancer (113). OA regulates aerobic glycolysis and tumor cell proliferation by downregulating HIF-1α, HK2, and PFK1, thereby suppressing glucose uptake and utilization, inhibiting cell growth, and reducing intracellular lactate levels (114).

### 3.2 Modulation of lipid metabolism

As noted above, lipid metabolism—including fatty acid, phospholipid, and cholesterol metabolism—plays a critical role in tumor development and progression. A number of natural products have been reported to exert anticancer effects by modulating key enzymes and signaling pathways involved in lipid metabolism. For example, both luteolin and resveratrol inhibit FASN. In HT-29 cells, luteolin downregulates anti-apoptotic proteins and induces cell cycle arrest (115). Resveratrol modulates lipid metabolism in cancer through multiple mechanisms, thereby restoring a metabolic balance more compatible with physiological demands. Specifically, resveratrol inhibits lipid synthesis via suppression of SREBPs, activates sirtuins in concert with AMPK activation, and downregulates the PI3K/AKT/mTOR pathway, ultimately leading to cancer cell apoptosis. Clinically, resveratrol has been shown to reduce tumor burden and metastasis by decreasing serum TAG, VLDL, and LDL levels in cancer patients, suggesting its potential role in modulating cancer initiation and progression (116). In addition, curcumin inhibits ACLY activity, thereby lowering acetyl-CoA levels, reducing lipid biosynthesis, and suppressing tumor growth (117). Similarly, berberine suppresses ACLY activity, contributing to the inhibition of tumor progression and metastasis (118). Furthermore, naringenin regulates cholesterol metabolism by reducing foam cell formation, decreasing intracellular cholesterol levels and esterification, and enhancing cholesterol efflux in THP-1 macrophages (119). Taken together, these findings highlight that natural products not only regulate glycolysis and mitochondrial function but also reprogram lipid metabolism, thereby exerting multifaceted anticancer effects through the coordinated targeting of multiple metabolic pathways.

### 3.3 Modulation of amino acid metabolism

Amino acid metabolism provides both metabolic substrates and regulatory signals critical for tumor growth. Distinct amino acid dependencies across cancers highlight therapeutic potential. Strategies involving amino acid depletion or supplementation show context-dependent antitumor effects. TCMs also regulate amino acid metabolism: Huangqin Tang (HQT) alleviates and delays colitis-associated cancer (CAC) by maintaining amino acid homeostasis and modulating the PI3K/AKT/mTOR pathway, thereby balancing proliferation and apoptosis (120). Early administration of Wu Mei Wan (WMW) prevents CAC development by suppressing myeloid-derived suppressor cells through regulation of amino acid metabolism and the PI3K/Akt pathway (121). The aqueous extract of Kudingcha inhibits the expression of glycerol-3-phosphate dehydrogenase, disrupts amino acid metabolism, induces mitochondrial oxidation in breast cancer cells, and the resulting ROS reverse the epithelial mesenchymal transition process (122). In addition, licorice root extract (123) and modified Si Jun Zi Tang (124) have been demonstrated, in both *in vitro* and *in vivo* studies, to regulate multiple metabolic pathways, including glycolysis and glutamine, serine, and glycine-related metabolism, thereby influencing cancer cell proliferation, apoptosis, and migration.

### 3.4 Targeting mitochondrial function and oxidative stress

Mitochondrial dysfunction plays a crucial role in tumor-induced immunosuppression. Aberrant mitochondrial metabolism and excessive reactive ROS production remodel the TME, thereby weakening effective antitumor immune responses (125). In addition, studies have revealed that certain natural compounds can induce tumor cell death by targeting mitochondrial function and enhancing oxidative stress. For instance, rhein–DCA accumulates in mitochondria, where it inhibits glycolysis through the PDK–PDH axis and disrupts the respiratory chain, thereby inducing oxidative stress, reducing lactate levels, and promoting cell death in CRC models (126). Frondoside A downregulates Bcl-2 and survivin, increases ROS production, and promotes cytochrome c release, ultimately triggering mitochondrial apoptosis c release, ultimately triggering mitochondrial apoptosis (127). Ginsenoside Compound K activates ROS-mediated mitochondrial apoptosis, leading to cytochrome c release and the activation of caspase-9/-3 (128). Resveratrol (RES) reprograms energy metabolism in colorectal cancer cells by enhancing oxidative phosphorylation, suppressing glycolysis and the pentose phosphate pathway, and increasing ATP production. In addition, it acts on the mitochondrial pyruvate dehydrogenase (PDH) complex to boost PDH activity, with calcium signaling and the CamKKB/AMPK pathway mediating the enhancement of glucose oxidation. These effects collectively shift cancer cell metabolism toward a more oxidative, energy-efficient state (129). In summary, natural products such as oleanolic acid, resveratrol, and curcumin embody the hallmarks of multi-target metabolic regulation by simultaneously regulating glycolysis, mitochondrial function and oxidative stress, lipid metabolism, and amino acid metabolism, highlighting their unique ability to reprogram tumor metabolism and exert comprehensive anticancer effects. This multi-target metabolic reprogramming highlights the therapeutic potential of natural compounds in comprehensive cancer intervention.

Overall, the natural products discussed in this section encompass both TCM formulas and single compounds, including their derived bioactive monomers. TCM formulas, such as HQT and WMW, achieve system-level regulation of tumor metabolic reprogramming through synergistic multi-component, multi-target, and multi-pathway effects, thereby inhibiting cancer cell growth, invasion, and migration. However, the complexity of these formulations, which often contain hundreds of chemical constituents, poses challenges for standardization and mechanistic elucidation. In contrast, single compounds like curcumin and resveratrol demonstrate well-defined antitumor immunomodulatory activities by modulating immune cell activation, functional maintenance, and cytokine production. Due to their clearly characterized chemical structures and target specificity, these monomeric compounds offer distinct advantages for mechanistic studies of tumor metabolism and immune regulation.

## 4 Natural products in cancer immunotherapy: metabolic reprogramming, immune activation, and advanced delivery strategies

Accumulating evidence indicates that natural products exert broad immunomodulatory effects making them promising adjuvants in cancer immunotherapy. Within the tumor microenvironment, natural compounds can reprogram dysregulated immune cell metabolism, restore antitumor immune function, and alleviate immunosuppressive signaling. Moreover, many natural products enhance immune activation by inducing immunogenic cell death and modulating immune checkpoint pathways, thereby improving responses to immune checkpoint inhibitors. However, their clinical translation is often limited by poor bioavailability and inadequate tumor targeting. Recent advances in delivery technologies, particularly nanotechnology-based systems, provide effective strategies to enhance stability, targeting efficiency, and therapeutic efficacy.

### 4.1 Natural products remodel tumor microenvironment through immune cell metabolic reprogramming

With increasing insights into the mechanisms by which natural products modulate metabolism in cancer, studies have found that these natural products have the potential to reshape the TME by reprogramming immune cell metabolism and restoring antitumor immunity.

As mentioned above, RES exerts multi-target metabolic regulatory effects in cancer cells. Moreover, current study showed that RES directly reprograms T cell metabolism in the TME. Low-dose RES (20 µM) downregulates GLUT1, decreasing glucose uptake, glycolysis, lactate production, and extracellular acidification, while enhancing glutamine metabolism through upregulation of ASCT2 and GLS2. These changes shift energy metabolism from glycolysis toward OXPHOS, increase ATP levels and mitochondrial ROS, and activate p53 via ATR-dependent stress signaling. Importantly, these metabolic effects enhance IFN-γ secretion and T cell effector functions, demonstrating that RES-mediated metabolic reprogramming can strengthen antitumor immunity (130, 131). Clinical studies investigating resveratrol in cancer remain limited but span multiple tumor types, including colorectal, liver, breast, and colon cancers, as well as multiple myeloma and other solid tumors. Early-phase trials (e.g., NCT00433576, NCT00256334) demonstrated acceptable safety and tissue bioavailability at moderate doses, whereas high-dose formulations such as SRT501 in multiple myeloma (NCT00920556) caused renal toxicity, highlighting dose-related safety concerns. Most studies focused on pharmacokinetics, biomarker modulation, and metabolic effects rather than therapeutic efficacy. These findings suggest that while resveratrol exhibits promising immunometabolic regulatory potential, further well-designed randomized clinical trials are required to establish its clinical benefits in oncology (**Table 2**).

**Table 2:** Summary of Clinical Trials Investigating Resveratrol in Cancer and Tumor-Related Conditions.

NCT number	Study title	Study status	Conditions	Interventions
NCT0092080	A Clinical Study to Assess the Safety, Pharmacokinetics,and Pharmacodynamics of SRT501 in Subjects With Colorectal Cancer and Hepatic Metastases	COMPL-ETED	Neoplasms,Colorectal	DRUG: Placebo| DRUG: SRT501
NCT01476592	A Biological Study of Resveratrol’s Effects on Notch-1 Signaling in Subjects With Low Grade Gastrointestinal Tumors	COMPL-ETED	Neuroendo-crine Tumor	DIETARY_SUPPLEMENT: Resveratrol
NCT00256334	Resveratrol for Patients With Colon Cancer	COMPL-ETED	Colon Canc-er| Cancer	DRUG: Resveratrol
NCT00098969	UMCC 2003–064 Resveratrol in Preventing Cancer in Healthy Participants	COMPL-ETED	Unspecified Adult Solid Tumor, Protocol Specific	DRUG: resveratrol
NCT02261844	Resveratrol and Human Hepatocyte Function in Cancer	WITHD-RAWN	Liver Cancer	DIETARY_SUPPLEMENT: Resveratrol|DRUG: Placebo
NCT03253913	Resveratrol and Sirolimus in Lymphangioleiomyomatosis Trial	COMPL-ETED	Lymphangioleiomyomatosis	DRUG: Sirolimus|DRUG: Resveratrol
NCT00920556	A Clinical Study to Assess the Safety and Activity of SRT501 Alone or in Combination With Bortezomib in Patients With Multiple Myeloma	TERMI-NATED	Multiple Myeloma	DRUG: 5.0g SRT501|DRUG: Bortezomib
NCT00433576	Resveratrol in Treating Patients With Colorectal Cancer That Can Be Removed By Surgery	COMPL-ETED	Adenocarcinoma of the Colon|Adenocarcinoma of the Rectum, Stage I Colon Cancer, Stage I Rectal Cancer, Stage II Colon Cancer, Stage II Rectal Cancer, Stage III Colon Cancer, Stage III Rectal Cancer	DRUG: resveratrol|OTHER: pharmacological study|OTHER: laboratory biomarker analysis
NCT00578396	Phase I Biomarker Study of Dietary Grape-derived Low Dose Resveratrol for Colon Cancer Prevention	WITHD-RAWN	Colon Cancer	DIETARY_SUPPLEMENT: grapes|DIETARY_SUPPLEMENT: grapes|DIETARY_SUPPLEMENT: grapes
NCT03482401	Disposition of Dietary Polyphenols and Methylxanthines in Mammary Tissues From Breast Cancer Patients	COMPL-ETED	Breast Cancer	DIETARY_SUPPLEMENT: Polyphenol

Data were retrieved from ClinicalTrials.gov (https://clinicaltrials.gov) as of October 15, 2025.

Furthermore, research by Carrasco-Pozo C et al. (132), demonstrated that gut microbiota metabolites derived from dietary polyphenols (e.g., quercetin, proanthocyanidins, and kaempferol), such as 3,4-dihydroxyphenylacetic acid (3,4-DHPAA) and 4-hydroxyphenylacetic acid (4-HPAA), can counteract the immunosuppressive effects of heme on macrophages. Heme induces an anti-inflammatory phenotype via HO-1, suppresses glycolysis, reduces IP-10 production, and impairs macrophage phagocytosis and tumor cell killing. Both 3,4-DHPAA and 4-HPAA restore glycolytic activity, upregulate enzymes involved in glycolysis and the pentose phosphate pathway, and stabilize HIF-1α, thereby recovering macrophage inflammatory responses, phagocytosis, and cytotoxicity toward tumor cells (133). In addition, although some studies have not directly explored metabolic regulation within the TME, they have explored how natural products can improve immune cell metabolism and thus affect immune function. For example, studies have reported that dioscin promotes FAO in macrophages through the mTORC2/PPAR-γ pathway (134). Similarly, curcumin modulates lipid metabolism in THP-1-derived macrophages by upregulating lipid transport genes such as CD36/FAT and FABP-4, leading to lipid accumulation and potentially limiting lipid utilization by tumor cells, ultimately inhibiting tumor growth (135, 136).

Collectively, these findings highlight that natural products, either directly or via microbial metabolism, can reprogram immune cell metabolism-targeting glycolysis, the pentose phosphate pathway, and lipid metabolism-to restore immune activity and enhance antitumor responses within the TME.

### 4.2 Natural products enhance antitumor immunotherapy

Accumulating evidence indicates that natural products exert multifaceted immunomodulatory effects within the TME, thereby enhancing antitumor immunity and improving the efficacy of cancer immunotherapies (137). A growing number of natural compounds have been shown to induce immunogenic cell death (ICD), a process characterized by the exposure or release of damage-associated molecular patterns (DAMPs), such as calreticulin (CRT), high-mobility group box 1 (HMGB1), and heat shock proteins (HSPs) (138). To date, only a limited number of agents have been shown to induce ICD, yet accumulating evidence indicates that several natural products possess this capacity. Compounds such as capsaicin (139, 140), ginsenoside Rg3 (140), resveratrol (141), and the combination of quercetin with alantolactone (142)can trigger ICD by promoting the exposure or release of DAMPs, including calreticulin, heat shock proteins, and HMGB1. These events enhance dendritic cell uptake, maturation, and antigen presentation, leading to robust CD8^+^ T cell–mediated antitumor immune responses and long-term immune memory. In addition, some natural products, such as shikonin, function as potent adjuvants for dendritic cell–based tumor vaccines by further amplifying ICD-associated immunogenicity (143). Collectively, these findings demonstrate that natural products can convert dying tumor cells into “*in situ* cancer vaccines,” highlighting their potential as immune-enhancing agents in cancer immunotherapy.

In addition to enhancing immune priming, several natural products directly regulate immune checkpoint pathways and immunosuppressive networks within the TME. Compounds such as, baicalin (144), silibinin (145), panaxadiol (146), and Bu-zhong-yi-qi decoction (147) have been shown to downregulate PD-1 or PD-L1 expression through modulation of upstream signaling pathways, including JAK–STAT, NF-κB, and PI3K–AKT–mTOR signaling. Unlike monoclonal antibodies that physically block PD-1/PD-L1 interactions, these agents reprogram the immunosuppressive milieu by simultaneously affecting tumor cells, tumor-associated macrophages, regulatory T cells, and inflammatory cytokine networks. Consistent with these mechanisms, increasing preclinical evidence demonstrates that natural products can synergize with immune checkpoint inhibitors (ICIs) to overcome therapeutic resistance. For instance, andrographolide enhances anti–PD-1 efficacy by suppressing COX-2/PGE2–mediated immunosuppression (148), whereas diosgenin potentiates the activity of anti–PD-1 antibodies by promoting T cell infiltration through modulation of the gut microbiota (149). Moreover, Xu et al. demonstrated that puerarin enhances anti–PD-L1 efficacy by suppressing ROS production, thereby reducing CAF-mediated physical barriers, increasing T-cell infiltration, and alleviating immunosuppression in the tumor microenvironment (150).

Collectively, these findings underscore the unique capacity of natural products to modulate tumor–immune crosstalk at multiple levels, ranging from immune priming and checkpoint regulation to metabolic and microbial reprogramming. By virtue of their multi-target, low-toxicity, and system-level regulatory properties, natural products represent promising adjuvant agents for combination immunotherapy strategies aimed at enhancing efficacy, mitigating resistance, and improving long-term clinical outcomes.

### 4.3 Advanced delivery systems for natural products

Recent advances in nanotechnology-based drug delivery systems have provided powerful solutions to the long-standing translational barriers of natural products, particularly poor solubility, limited bioavailability, and non-specific tissue distribution. A wide range of nano-drug delivery systems (NDDS), including lipid nanoparticles, nanoliposomes, polymeric micelles, nanosuspensions, and self-microemulsifying systems, have been developed to enhance the pharmacokinetic stability and therapeutic efficacy of bioactive natural compounds (151). These nanoformulations improve aqueous solubility, protect labile molecules from degradation, prolong systemic circulation, and enable passive or active tumor targeting via the enhanced permeability and retention (EPR) effect or ligand-mediated delivery (152). Moreover, nano-delivery platforms enable co-encapsulation of natural products with chemotherapeutic or immunotherapeutic agents, facilitating synergistic immunomodulatory effects and improving responses to immune checkpoint blockade (153).

For instance, Curcumin exhibits poor stability under physiological conditions, limiting its clinical translation. Chen et al. developed a nanoencapsulated formulation that achieved targeted delivery while improving chemical stability (154). Besides monotherapy, nanoplatforms also facilitate synergistic combination drug strategies. Zhang et al. co-encapsulated docetaxel and resveratrol in liposomes, achieving synergistic antitumor efficacy against prostate cancer by simultaneously inducing tumor cell apoptosis and regulating the tumor microenvironment (153). Xiao et al. reported a novel nanoparticle system functionalized with anti–PD-1 antibodies and loaded with curcumin, featuring dual pH responsiveness. Compared with free curcumin, this nanoformulation significantly enhanced cellular uptake. In addition to benefiting from the EPR effect, surface conjugation of anti–PD-1 antibodies facilitated specific binding of the nanoparticles to PD-1^+^ T cells. Owing to their pH-sensitive properties, these nanoparticles exhibited increased tumor selectivity, enabling greater intratumoral delivery of curcumin. This dual-delivery strategy effectively co-localized anti–PD-1 antibodies and curcumin within tumors and demonstrated superior therapeutic efficacy in both *in vitro* and *in vivo* models (155).Collectively, these technological advances highlight nanotechnology-based delivery as a critical and clinically relevant strategy to bridge the gap between the promising immunometabolic activities of natural products and their successful translation into cancer immunotherapy.

## 5 Conclusion and future perspectives

Natural metabolism-regulating agents have emerged as promising adjuvant strategies in cancer immunotherapy due to their ability to reprogram metabolic pathways and restore antitumor immunity. Notably, the complex composition of traditional Chinese medicine warrants careful consideration when used in combination with immunotherapy, as excessive immune activation may trigger immune-related adverse events, and certain traditional Chinese medicines with hepatotoxic potential may increase the risk of organ injury during combination treatment. However, clinical translation remains challenging, with key issues including dose optimization, safety evaluation, and patient selection. Although several natural compounds have entered preclinical or early clinical trials, the transition from bench to bedside is often hindered by limited pharmacokinetic stability, unclear mechanisms of action, and heterogeneous patient responses.

Looking ahead, precision targeting of specific metabolic pathways offers substantial therapeutic potential, particularly when integrated with advances in immunotherapy. Progress in this field will require close interdisciplinary collaboration among pharmacology, immunology, and metabolism research, as well as innovative approaches to identify novel compounds, such as immune-sensitizing agents from traditional Chinese medicine. Future studies should focus on elucidating the intricate interplay between metabolism and the immune system to design more selective and effective therapeutic strategies. Addressing translational barriers and validating natural products in rigorous clinical settings will be essential steps toward realizing their clinical application and therapeutic promise.
